# Integration of Water, Sanitation, and Hygiene for the Prevention and Control of Neglected Tropical Diseases: A Rationale for Inter-Sectoral Collaboration

**DOI:** 10.1371/journal.pntd.0002439

**Published:** 2013-09-26

**Authors:** Matthew C. Freeman, Stephanie Ogden, Julie Jacobson, Daniel Abbott, David G. Addiss, Asrat G. Amnie, Colin Beckwith, Sandy Cairncross, Rafael Callejas, Jack M. Colford, Paul M. Emerson, Alan Fenwick, Rebecca Fishman, Kerry Gallo, Jack Grimes, Gagik Karapetyan, Brooks Keene, Patrick J. Lammie, Chad MacArthur, Peter Lochery, Helen Petach, Jennifer Platt, Sarina Prabasi, Jan Willem Rosenboom, Sharon Roy, Darren Saywell, Lisa Schechtman, Anupama Tantri, Yael Velleman, Jürg Utzinger

**Affiliations:** 1 Department of Environmental Health, Emory University, Atlanta, Georgia, United States of America; 2 Children Without Worms, Taskforce for Global Health, Atlanta, Georgia, United States of America; 3 International Trachoma Initiative, Taskforce for Global Health, Atlanta, Georgia, United States of America; 4 Bill & Melinda Gates Foundation, Seattle, Washington, United States of America; 5 Save the Children, Washington, D.C., United States of America; 6 Hubert Department of Global Health, Emory University, Atlanta, Georgia, United States of America; 7 Faculty of Infectious and Tropical Diseases, London School of Hygiene & Tropical Medicine, London, United Kingdom; 8 Millennium Water Alliance, Washington, D.C., United States of America; 9 Department of Epidemiology, University of California-Berkeley, Berkeley, California, United States of America; 10 The Carter Center, Atlanta, Georgia, United States of America; 11 Schistosomiasis Control Initiative, Imperial College, London, United Kingdom; 12 WASH Advocates, Washington, D.C., United States of America; 13 Department of Civil and Environmental Engineering, Imperial College, London, United Kingdom; 14 World Vision, Washington, D.C., United States of America; 15 CARE International, Atlanta, Georgia, United States of America; 16 Taskforce for Global Health, Atlanta, Georgia, United States of America; 17 Center for Global Health, United States Centers for Disease Control and Prevention, Atlanta, Georgia, United States of America; 18 Helen Keller International, New York, New York, United States of America; 19 United States Agency for International Development, Washington, D.C., United States of America; 20 ORBIS International, New York, New York, United States of America; 21 Waterborne Disease Prevention Branch, United States Centers for Disease Control and Prevention, Atlanta, Georgia, United States of America; 22 Plan International, Washington, D.C., United States of America; 23 WaterAid America, Washington, D.C., United States of America; 24 Sabin Vaccine Institute, Washington, D.C., United States of America; 25 WaterAid UK, London, United Kingdom; 26 Swiss Tropical and Public Health Institute, Basel, Switzerland; 27 University of Basel, Basel, Switzerland; University of Florida, United States of America

## Abstract

Improvements of water, sanitation, and hygiene (WASH) infrastructure and appropriate health-seeking behavior are necessary for achieving sustained control, elimination, or eradication of many neglected tropical diseases (NTDs). Indeed, the global strategies to fight NTDs include provision of WASH, but few programs have specific WASH targets and approaches. Collaboration between disease control programs and stakeholders in WASH is a critical next step. A group of stakeholders from the NTD control, child health, and WASH sectors convened in late 2012 to discuss opportunities for, and barriers to, collaboration. The group agreed on a common vision, namely “Disease-free communities that have adequate and equitable access to water and sanitation, and that practice good hygiene.” Four key areas of collaboration were identified, including (i) advocacy, policy, and communication; (ii) capacity building and training; (iii) mapping, data collection, and monitoring; and (iv) research. We discuss strategic opportunities and ways forward for enhanced collaboration between the WASH and the NTD sectors.

## Introduction

The prevention, control, and eventual elimination of many neglected tropical diseases (NTDs) depend heavily on the availability of improved water, sanitation, and hygiene (WASH) in endemic communities, as summarized in Table 1. Treatment alone will not break the cycle of transmission; improvements of WASH infrastructure and appropriate health-seeking behavior are essential to achieving sustained control, elimination, or eradication of many NTDs [1]–[3]. The global strategies for controlling and eliminating several NTDs, such as trachoma, schistosomiasis, soil-transmitted helminthiasis, and Guinea worm, specifically reference the need for improved water and sanitation. Yet, in practice, the repeated large-scale administration of antibiotics or anthelmintic drugs to at-risk populations [4], [5] is the primary focus of many NTD control programs. With this in mind, there is a pressing need to (i) identify best practices and build a strong evidence-base for collaborative programming and (ii) identify the most effective, sustainable, and scalable methods of integrating WASH and NTD control activities.

**Table 1:** WASH-related NTDs and transmission mechanisms.

Disease	Link Aspect	Transmission, Control, and Prevention
**Trachoma** a	Sanitation, hygiene, and water	Transmission between infected persons through direct contact or flies. Prevention: promotion of face washing of children, improved access to clean water, and proper sanitation for disposal of human waste to reduce fly population and transmission.
**Soil-transmitted helminthiasis** (ascariasis, hookworm, trichuriasis, and strongyloidiasis)^b^	Sanitation and hygiene	Eggs ingested through contaminated food or water, or directly by children with dirty hands or placing soil in mouth; hookworm larvae penetrate skin when walking barefoot on contaminated soil (no direct person-to-person transmission). Prevention through improved sanitation and hygiene (hand washing).
**Schistosomiasis** c	Sanitation, water, and hygiene	Infection: eggs of worms in human feces or urine contaminating water where emerging larvae enter freshwater snails. After development in snail, larval forms emerge in water and penetrate skin during contact with infested water. Control measures: snail control, improved sanitation and health education, reduced contact with surface water, and chemotherapy.
**Dracunculiasis** (Guinea worm)^d^	Water quality	Transmitted through ingestion of parasite-infected water fleas in contaminated water. Control measures: water source protection (protected wells/bore holes, treatment of contaminated water sources with insecticide, temephos, and water filtration; case containment; health education, surveillance, and reporting).
**Lymphatic filariasis** e	Sanitation (prevention) and hygiene (treatment)	Parasites transmitted by mosquitoes. Poorly constructed latrines and standing water increase presence of lymphatic filariasis-transmitting mosquito vectors. Patients with chronic disabilities are advised to maintain rigorous hygiene and take necessary precautions to prevent secondary infection; availability of water for limb washing important in reducing severity of lymphatic filariasis and good water management and sanitation can decrease mosquito breeding sites.
**Dengue** f	Water storage management	Virus transmitted by mosquitoes. Mosquito control: covering, emptying, and frequent cleaning of domestic water storage containers; applying appropriate insecticides to water storage outdoor containers. Epidemic control through insecticide spraying.
**Onchocerciasis** g	Water resource management	Parasite transmitted by blackfly in riverside locations. Measures for blackfly control: insecticide treatment of larval breeding sites (fast flowing water) but including water-flow manipulation if possible (dam sites, spillways).

Table adapted from „WASH: the silent weapon against NTDs” [36]. Table represents the strength of connection between, and potential impact of, WASH and disease, starting at the top, where WASH impact on disease is likely to be strongest and moving to the bottom of the table, where impact may be weakest.

a World Health Organization, “Prevention of blindness and visual impairment” 2012. http://www.who.int/blindness/causes/priority/en/index2.html (accessed: 5 August 2012).

b World Health Organization, “Intestinal worms” 2012. http://www.who.int/intestinal_worms/en/ (accessed 5 August 2012); World Health Organization, “Neglected tropical diseases” 2012. http://www.who.int/neglected_diseases/diseases/strongyloidiasis/en/ (accessed: 5 August 2012).

c World Health Organization, “Schistosomiasis: fact sheet no. 115” 2012. http://www.who.int/mediacentre/factsheets/fs115/en/index.html (accessed: 5 August 2012).

d World Health Organization, “Drancunculiasis: fact sheet no. 359” 2012. http://www.who.int/mediacentre/factsheets/fs359/en/index.html (accessed: 5 August 2012).

e World Health Organization, “Lymphatic filariasis: fact sheet no. 102” 2012. http://www.who.int/mediacentre/factsheets/fs102/en/index.html (accessed: 5 August 2012).

f World Health Organization, “Dengue and severe fever: fact sheet no. 117” 2012. http://www.who.int/mediacentre/factsheets/fs117/en/index.html (accessed: 5 August 2012).

g World Health Organization, “Priority eye diseases” 2012. http://www.who.int/blindness/causes/priority/en/index3.html(accessed: 5 August 2012).

In December 2012, some 30 WASH and NTD experts convened for a two-day roundtable discussion in Seattle, organized by Emory University and the Task Force for Global Health and hosted by the Bill & Melinda Gates Foundation. The objective of the discussion was to bring together thought leaders and to discuss opportunities to foster collaboration between WASH and NTD experts. The current Policy Platform article, compiled by a core writing team and finalized and vetted by all the participants of the roundtable, describes the proceedings and key outcomes of the two-day discussion. In the current piece, we first present background and current evidence for overlap between the WASH and NTD sectors (for a definition of the two sectors, see Table 2). Then we summarize identified priorities for collaboration and propose a common vision for specific collaborations between the two sectors arising from these priorities. Toward the end of the article, we outline the challenges for collaboration that must be addressed to ensure sustained and mutual benefit, and describe strategic opportunities proposed at the roundtable, readily grouped into four key thematic areas. We will conclude our piece with suggestions for practical next steps and ways forward between the two sectors.

**Table 2:** Definitions of WASH and NTD sectors.

Sector	Description	Primary Implementing and Advocacy Stakeholders	Primary Funding Stakeholders
**NTD control**	- The NTD sector consists of stakeholders in multi-lateral organizations, national and district level governments, NGOs, donors, and pharmaceutical companies that contribute directly to the treatment and prevention of the NTDs in order to achieve global elimination and control targets, in addition to decreasing morbidity and mortality among key risk groups in the short term.- The primary focus of much of the NTD sector has been the periodic distribution of drugs to at-risk groups to prevent morbidity.	- WHO- National Ministries of Health and Education- Multi-lateral organizations and NGOs- Pharmaceutical companies- Bi-lateral governments and aid agencies	- Pharmaceutical companies and International NGOs- National and district level governments- Foundations and international donors
**WASH**	- The WASH sector is a diverse group of stakeholders that includes national governments, international, and local NGOs, and multi-laterals, that contribute directly to increasing coverage of water, sanitation, and hygiene education in global, national, or sub-national areas with insufficient access.- Stated objectives are also diverse, but encompass the fulfillment of the human right to water and sanitation, in addition to improving health, education, gender equity, and economies.- Funding comes primarily from national governments and bilateral donors.	- Bi-lateral governments and aid agencies- Development banks- National Ministries of Public Works, Water Resources or Environment, Health, Education, and Finance- Multi-lateral organizations- Communities and users	- District level governments WASH-focused NGOs- NGOs and local community-based organizations

## Integration of WASH and NTDs

The World Health Organization's (WHO) 2020 goals for NTD control emphasize the need for both treatment and prevention of the NTDs. However, as pointed out by the editors of *The Lancet*, in response to the launch of the WHO Roadmap titled “Accelerating work to overcome the global impact of neglected tropical diseases: a roadmap for implementation” [6], improvements to WASH were listed last of the five strategies for addressing NTDs, and no specific targets or approaches to address this issue were discussed [7]. Nonetheless, some success at integration of WASH interventions into NTD control programs has been achieved among individual disease control strategies. Of particular note is the trachoma control program [8], for which the WHO endorses the “SAFE” strategy (Surgery to correct advanced stages of trachoma; Antibiotics to treat active infection; Facial cleanliness to reduce disease transmission; and Environmental change including increased access to water and improved sanitation). Hence, the SAFE strategy explicitly includes improved access to, and use of, water, sanitation, and hygiene – through either improvements in delivery, specific interventions, or both – and new research documents ancillary benefits of these improvements on other NTDs [9].

Though the NTD control community has explicitly recognized the importance of WASH for the prevention and control of NTDs [10], tangible improvement to WASH services on a global scale is primarily achieved by WASH sector investment, particularly from government stakeholders and, to a lesser extent, bi-lateral and multi-lateral donors *via* non-governmental organizations (NGOs) or direct government budget support. It stands to reason that collaboration with the WASH sector is the most efficient route to improving WASH services in disease-endemic areas. While there have been notable efforts to incorporate WASH infrastructure and messaging into NTD control programs, there has historically been little collaboration between NTD control programs and the WASH sector at either the national or international levels [11], [12]. From a health- and rights-based perspective, WASH and NTD donors and civil society organizations are equally concerned with the poorest and most marginalized populations, who typically have limited access to basic infrastructure and services, including water and sanitation, disproportionately high disease burden, and little access to education [2], [3]. Of course, there are other drivers for development funds, and historically, resources for water supply and sanitation improvements from development banks and governments have tended to focus on urban constituencies and systems, rather than on-site solutions for rural and marginalized populations [13]. For both sectors, and for the populations they seek to serve, there are, arguably, several clear opportunities and mutually reinforcing advantages to collaboration. These opportunities include advocacy, joint programming for greater efficiency and cost-effectiveness, and the sharing of best practices, all of which contribute to increased impact on the health and welfare of communities. However, past and present collaboration between the WASH and NTD sectors at various levels has met with mixed results, and there are numerous important challenges and obstacles to consider [14]. As a notable example, the attempted collaboration between the Guinea Worm Eradication Program and WASH sector stakeholders in NGOs, multi-laterals, and governments to ensure the construction of boreholes in Guinea worm endemic communities in Ghana and South Sudan demonstrated that differences in operational timelines, budgets, and targeting criteria were significant challenges to collaboration [15]–[17].

## Current Evidence

Although there is biological plausibility and a growing body of evidence suggesting impacts of WASH improvement on several NTDs [18]–[20], there is a paucity of rigorous experimental evidence. This knowledge gap makes it difficult to draw conclusive inferences and quantify the precise impact of WASH interventions on transmission and control of NTDs in general and in specific contexts, or to recommend which interventions should be prioritized or targeted to best contribute to disease control. A systematic review and meta-analysis established a correlation between soil-transmitted helminth infections and sanitation. Access to, and use of, sanitary facilities was correlated with lower odds of soil-transmitted helminth infection, including 0.54 (95% confidence interval [CI] 0.43–0.69) for *Ascaris lumbricoides*, 0.58 (95% CI 0.45–0.75) for *Trichuris trichiura*, and 0.60 (95% CI 0.48–0.75) for hookworm [21]. However, no randomized controlled trials were found, and the strength of the available data was limited. There are currently no similar reviews assessing the correlation between soil-transmitted helminthiasis and water access or hygiene. Two Cochrane reviews revealed only very few experimental studies assessing face washing [22], environmental sanitation, including access to household latrines and elimination of open defecation, or fly control [23] on trachoma, though these interventions are widely acknowledged to have some impact. No recent review has been conducted on WASH and schistosomiasis; the last one dates back more than 20 years [18]. Moreover, the evidence base describing specific WASH interventions with the greatest effect on NTDs remains to be established. From the NTD control perspective, more specific information about which WASH interventions are effective at mitigating exposure and reinfection, and how WASH interventions act in concert with mass drug administration (MDA), is needed.

## Identifying Priorities

The objectives of the Seattle two-day roundtable discussions were to identify common goals, acknowledge past challenges, identify barriers to working together, discuss ways to overcome challenges and barriers, and identify practical opportunities for collaboration and mutual benefit. Attendees included researchers, practitioners, donors, and representatives from advocacy, relief, and development NGOs. This dialogue was one in an ongoing series of discussions between the WASH and NTD sectors in 2012 to identify opportunities for engaging jointly to articulate and ensure progress toward common goals (Table 3).

**Table 3:** Recent meetings to discuss NTD-WASH sector collaboration.

Meeting	Venue, Location	Date
NTD Non-Governmental Development Organization Network (NNN) meeting [36]	Sydney, Australia	4–6 September 2012
Symposium: WASH and the NTDs: bridging the divide between treatment and prevention programs to reduce prevalence of NTDs	Water and Health Conference in Chapel Hill, United States of America	1 November 2012
Symposium: Beyond mass drug administration to sustained control of schistosomiasis and soil-transmitted helminthiasis: water, sanitation, and hygiene interventions	61^st^ annual conference of the American Society of Tropical Medicine and Hygiene, Atlanta, United States of America	12 November 2012

## Common Vision and Mutual Benefit for Collaboration between Sectors

Successful collaboration between the WASH and NTD communities depends on commitment toward a shared vision between the two sectors, as well as measurable benefits toward each sector's principal goals and measurements of success. The roundtable group defined a common long-term vision:

*Disease-free communities that have adequate and equitable access to water and sanitation, and that practice good hygiene.*

In order to sustain collaboration, benefits for both sectors must be clearly articulated. While the NTD sector is dependent on WASH improvement for the prevention, sustained control, or elimination of many NTDs, the WASH sector is not mutually dependent on the NTD sector to achieve its primary programmatic or outcome goals, which tend to relate to universal coverage of water supply and sanitation services. WASH organizations rarely target, measure, or justify their work based on disease control, though the health focus of WASH has important policy and advocacy considerations. Many WASH organizations emphasize a rights-based approach, which limits the need for health targets as primary goals [24]. One important consideration in the way in which we discuss collaboration is that, unlike the NTD sector that has largely coalesced around the WHO 2020 targets, stakeholders within the WASH sector are numerous and quite varied in their approaches, funding streams, objectives, and operations. These stakeholders include national and district governments, primary and small-scale service providers, NGOs, community organizations, policy makers, and bilateral donors. Generalizations about these various stakeholders are exceedingly difficult (Table 2).

## Challenges to Collaboration and Coordination

Efforts to integrate, collaborate, and coordinate the NTD and WASH sectors are not new [11]. Key stumbling blocks for collaboration include differences in the scale of interventions, indefinite timelines for WASH investments, and community engagement on one hand, and a large disparity between costs of WASH services in comparison to a primarily treatment-based control approach. MDA is usually coordinated at the national level, and requires intense periods of community mobilization for short, punctuated periods of time throughout the year. Contrarily, WASH interventions most often occur at the district or community levels, and require consistent engagement and funding from national, district, civil society, and household levels for entire life cycles of services. As a result, and because of the need for improved infrastructure, in the short-term, the cost of WASH implementation is exponentially higher than that of drug distribution, and the costs are often diffused over a number of stakeholders. This leads to incongruences in service provision over both space and time; in the WASH sector, the majority of funds are still spent in urban areas on reticulated infrastructure projects, rather than small-scale solutions or infrastructure in rural and peri-urban areas, where the burden of NTDs is highest and WASH access is lowest [13]. From the perspective of political leverage, the NTD community has, until recently, operated on a small budget in comparison with the WASH sector, and so may have had little resulting leverage to influence WASH donors, organizations, and government stakeholders in targeting WASH activities to NTD-endemic areas.

Additionally, both sectors are undergoing transition. The NTD community has more recently coalesced around integrated NTD programming, and control and elimination targets, rather than within disease-specific silos, but harmonization of approaches and key messages is still nascent [25], [26]. The WASH sector has traditionally been hardware-focused and closely aligned with Ministries of Water and Public Works, but has been transitioning to a greater focus on long-term financing, governance, and behavior change interventions related to hand-washing and sanitation, for which clear metrics have yet to be fully defined. Mapping and district-level data collection have been useful tools in both the NTD and WASH sectors, and joint mapping and shared data collection present valuable opportunities for cost savings and informing both sectors toward common goals. However, harmonizing scales and sampling frames, as well as understanding and conveying the breadth of data already collected, are clear challenges.

The perceived imbalance of need, alluded to in the previous section, is clearly articulated in the following challenge posed by several WASH expert participants: Why should the WASH sector implement in areas designated by the NTD sector, rather than other marginalized areas? Participants agreed that a funding flow from the sponsors of NTD programs, and advocacy to support relevant parts of the WASH sector, might be necessary to encourage such coordination. Ultimately, however, to sustain collaboration, mutual goals and metrics must be institutionalized in global targets, national strategies, and funding streams. Such a shift is unlikely to be achieved in time for the 2020 NTD targets, and defining a realistic and sustained role for WASH in NTD control efforts will be necessary.

## Strategic Opportunities for Collaboration, Coordination, and Consultation

Participants recognized that full-scale collaboration and sectoral integration is difficult (requires harmonization of goals, metrics, and implementation) and may not be immediately realistic within current policy frameworks. However, they identified opportunities for targeted collaboration, coordination, and consultation in key areas, on specific projects, and in strategic countries. A multitude of short-, medium-, and long-term opportunities were identified within each strategic focus, and within current policy frameworks and program limitations. Through a grounded discussion of sector needs, four key areas of focus have been developed and ways forward were discussed for each of them: (i) advocacy, policy, and communication; (ii) capacity building and training; (iii) mapping, data collection, and monitoring; and (iv) research. Below, we summarize the potential short-term wins and opportunities in each key area, as well as realistic next steps to facilitating those opportunities.

### Advocacy, Policy, and Communication

The purpose of improved advocacy and communication is to leverage support from both sides to engage policy-makers and donors, and create common platforms for dialogue, harmonized messages, and shared knowledge on issues of joint concern for WASH and NTD programs. Improved advocacy and communication that builds on, and speaks to, donor priorities such as value-for-money, measurable impact, and improved efficiency across programs can help to influence donors and implementers in the WASH and NTD control sectors, and improve the enabling environment for cross-sectoral collaboration. WASH organizations' ability to target implementation is often limited by donor restrictions, suggesting that advocacy toward donors to raise awareness of the integral connection between WASH and NTDs may create greater targeting flexibility at the WASH implementation level.

In endemic countries, advocacy at the government level can help to increase ownership of combined approaches to WASH and NTD control, and underscore the need for national strategic plans. Government ministries are the key stakeholders in delivering both MDA and WASH services, but accountable government departments and key collaborators for WASH and NTD control are most often different ministries (typically the Ministries of Health and Education for NTD control, and Ministries of Water, Environment, Public Works, Health, and Education for WASH). Advocacy could be targeted at developing linkages at the national and district levels in strategic countries that have both strong capacity for MDA and existing explicit commitments to universal access to water and sanitation. Advocacy that raises awareness among governments of the burden of NTDs and WASH-related diseases in the context of higher-priority issues such as malaria, tuberculosis, and HIV/AIDS may help to elevate both issues in national importance. Furthermore, advocating among both donors and government stakeholders for proven platforms, such as school health and nutrition approaches, may generate successful programming and partnerships at the implementation level that can then be rapidly scaled up.

**Figure 1:**
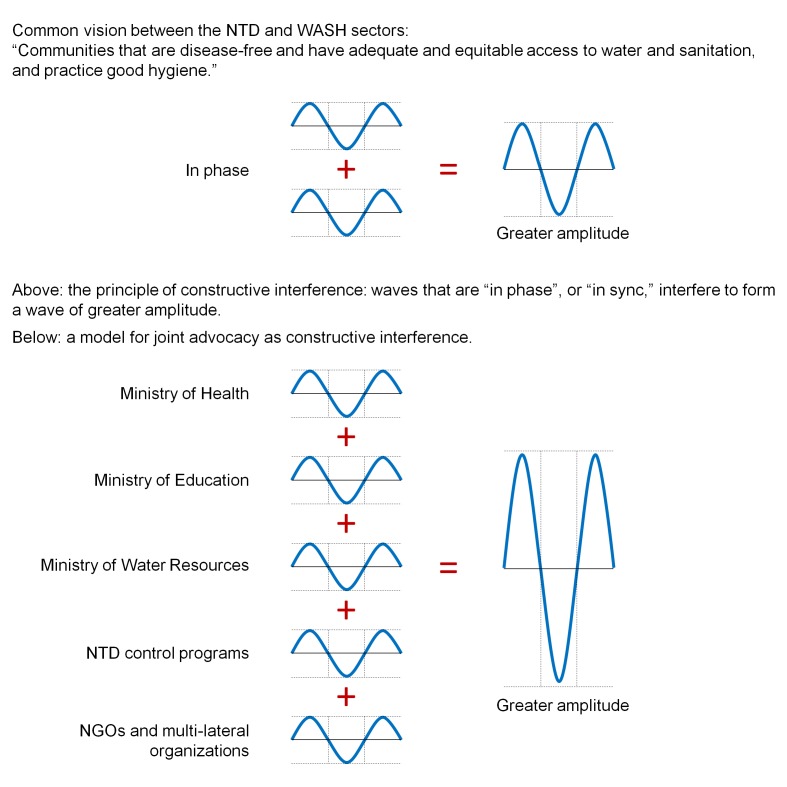
Joint advocacy and harmonized messaging between WASH and NTD sectors as “constructive interference.” Figure adapted from http://www.physicsnet.co.uk [37].

The importance and impact of harmonized messages to communities and donors were also recognized as essential. Harmonized messages from the WASH and NTD control sectors to donors and government stakeholders can amplify the appeal to WASH and NTD collaboration. Harmonized messages to communities can ensure that key messages are not minimized by “destructive interference,” whereby too many out-of-sync messages lead to an overall obfuscation and decrease in intensity. Contrarily, joint advocacy and harmonized messages among WASH and NTD partners can create “constructive interference,” whereby the assertion for greater collaboration, and key messages in hygiene and sanitation behavior change, are strengthened by stakeholder voices in concert (for an illustration of this concept, see Figure 1).

Next steps identified by the meeting participants pertaining to advocacy, policy, and communication are summarized in Table 4.

**Table 4:** Opportunities and next steps in advocacy for WASH and NTD sectors.

Develop coherent and aligned WASH and NTD messages for specific audiences: create a working group to conduct an inventory of existing material and gaps, and create an advocacy action plan
Better engage with pharmaceutical companies: include WASH/NTD information in calls and reports to donors
Develop a congressional briefing on WASH/NTDs
Reach out to non-traditional donors and donors across WASH and NTD sectors
Pilot demand generation strategies for collaborative WASH and NTD programming in 2–3 countries
Reframe the needs between WASH and NTD sectors based on cost and impact, and communicate appropriate evidence base
Create a list of messages linking appropriate research that donors would like to be able to impart
Provide programmatic guidance to USAID-WASH and other organizations; increase human resources capacity for advocacy of improved WASH services alongside NTD control in endemic countries

### Capacity Building and Training

The purpose of improved capacity building and training is to address knowledge gaps among stakeholders from each sector and to create effective information exchange mechanisms between the WASH and NTD sectors in order to enable more informed collaboration. In many cases, both sectors utilize similar messaging and shared communications objectives, particularly related to hygiene and sanitation behaviors (for an example, see: http://www.freshschools.org/pages/default.aspx). Collaboration to ensure harmonized messages can reduce conflicting messages and contribute to amplified communication for long-term behavior change. In some cases, it may be relatively easy to integrate NTD control messages into existing WASH sector approaches, such as by incorporating messages about face-washing to prevent trachoma into hygiene education programs, alongside hand-washing messages.

Cross-sector dialogue at the UNC Water and Health Conference in October 2012 suggested that many in the WASH sector are not aware of NTDs and their connection with WASH. Hence, training WASH practitioners on NTDs, and increasing awareness of the issue within the WASH sector, may lead to increased coordination and greater recognition of collaborative opportunities. However, there is little existing training material oriented toward helping WASH sector partners better incorporate NTD control messages with existing and compatible WASH messages.

Updating, harmonizing, and sharing existing training materials will help to avoid redundancies and competing messages, as well as encourage cost savings in the development and distribution of training materials. Table 5 summarizes next steps identified by the group.

**Table 5:** Opportunities and next steps in capacity building and training.

Conduct an inventory of existing training materials to determine gaps and overlapping messages
Create an open-source format for sharing data, materials, resources, and research between the WASH and NTD sectors, and beyond
Co-produce and share resources between the sectors: e.g., organizations collaborate to author a single manual on WASH in schools
Establish national-level forums in endemic countries to identify specific needs and take advantage of existing WASH and NTD initiatives
Begin dialogue at the national level in select pilot countries to identify and address specific capacity gaps
In both sectors, revise indicators for success in capacity building and training to reflect quality, relevance, and integration with existing mechanisms and forums
Provide input into the creation and dissemination of WASH/NTD manuals currently being developed jointly by International Trachoma Initiative, Children Without Worms, and Emory University

### Mapping, Data Collection, and Monitoring

The purpose of improved mapping and monitoring in the NTD and WASH communities is to identify areas that are at greatest need of interventions, to ascertain the required frequency of treatment in light of WASH coverage and other risk factors, to track progress for achieving WHO and Millennium Development Goal (MDG) targets [27], and share costs of data collection at the ground level [28]–[30]. Both the WASH and NTD sectors conduct mapping and ground-level data collection, but currently, only a small amount of data collected are of shared value. Geographic and demographic targets for both sectors overlap, and shared or coordinated data collection presents an enormous opportunity for cost savings.

Mapping of NTD-endemic areas, particularly the delineation of high-risk areas for one or multiple NTDs, has contributed substantially to targeting of MDA and other interventions, and was a key feature in rolling out national-level control programs. Some effort has been made to include WASH indicators in current large-scale NTD mapping efforts, but without much consultation with the WASH community regarding appropriate indicators, and with substantial difficulties in synchronizing the sampling frames of each sector [30].

Mapping within the WASH sector has been largely done at the program level by NGOs or at the national level by surveys, such as the USAID demographic and health survey (DHS) or UNICEF multiple indicator cluster survey (MICS). These data have not typically been useful for thorough district or community-level program targeting. However, some WASH mapping tools are under development that can provide up-to-date information on water system functionality, which help to better identify access gaps and monitor sustainability (see FLOW at: www.waterforpeople.org/flow-mapping/; and WaterMapper at: www.waterpointmapper.org/).

Enhanced coordination, or at least consultation, among these efforts could leverage opportunities for targeting WASH sector activities in NTD-endemic areas. Additionally, more nuanced WASH mapping may contribute to posttreatment surveillance efforts; identifying areas where WASH inputs are scarce and coverage is insufficient could help target at-risk sites for ongoing monitoring. Global mapping of several NTDs is likely to be achieved in the next three to five years as 2020 targets approach, so the window of opportunity for coordinated mapping is open wide, but only for a few years.

Increased coordination in mapping and data collection may also help to elaborate more effective cross-sectoral impact indicators. With foresight, and attention to emerging research, new indicators should target the nexus between nutritional disorders, NTDs, and WASH interventions, and should include such measures as height-for-age (stunting), cognitive impairment, and others. These indicators would require attention to improvements in both WASH and NTD control. WASH interventions can make significant contributions, especially for MDG 4 target on reducing child mortality. The most commonly used indicator of WASH impact on child mortality has been the reduction of diarrheal disease in children under the age of 5 years. However, with new evidence linking under-nutrition with pathogenic environments, including helminths and intestinal protozoa, WASH's impact on child mortality may also be measurable *via* a reduction of the load of pathogens in children.

For both sectors, current mapping efforts are necessary only in the absence of functional management information systems at the national and international levels. However, both sectors should move toward systems with routinely collected, updated, and cross-linked data that are used by governments for planning and budgeting. With these information systems in place, water programs would be able to use health indicators to identify priority areas for service allocation, and the health sector could use WASH coverage indicators to identify risk areas for disease transmission. Short-term opportunities for coordination and consultation regarding mapping, data collection, and monitoring are summarized in Table 6.

**Table 6:** Opportunities and next steps in mapping, data collection, and monitoring.

Create a centralized resource for all available maps and data related to WASH and NTDs; e.g., a website to host mapping resources and links to sites where data already exists regarding WASH and NTDs separately or together
Compile a list of indicators currently used by the WASH and NTD control programs respectively, and determine gaps
Establish common indicators for WASH and NTDs, realistic to mapping efforts

### Research

The purpose of research cross-talks between the WASH and NTD sectors should be to inform advocacy; help to identify gaps, barriers, and technical obstacles to collaboration; and directly improve efficacy and impact of WASH and NTD control programs. Participants expressed a need for policy-relevant research that could address operational issues. One key example discussed was investigating the confluence of school-based MDA and school WASH and associated costs. Several comprehensive and collaborative school-based platforms that include both deworming and WASH access are well established and provide valuable arenas for research.

There is also a need for research that more rigorously describes the impact of actual WASH interventions on NTD control. Considerable empirical evidence suggests that improved WASH is associated with reduced trachoma, soil-transmitted helminthiasis, and schistosomiasis. However, the vast majority of studies are cross-sectional, and hence the evidence base is weak. Furthermore, much of the existing literature examines WASH as a monolithic and often infrastructure-oriented intervention, while actual WASH interventions are more nuanced and often address only a portion of total WASH improvement. A deeper understanding of the role of WASH on reinfection patterns, and associated cost-benefit of WASH interventions for NTD prevention, would be useful for policy and planning of MDA and for advocacy. Some suggested next steps identified in relation to research are summarized in Table 7.

**Table 7:** Opportunities and next steps in research.

Conduct a survey of WASH/NTD research priorities; publish findings in public domain to inform future research
Identify donors willing to support collaborative research
Match research questions with advocacy and program needs
Determine costs of WASH, and additive value for NTD control, nutrition, etc.
Adjust the quality of research to the audience/end use
Conduct more research regarding drivers of WASH behaviors and behavior change

### Cross-Cutting Opportunities

Several cross-cutting opportunities emerged in relation to geographic and thematic zones of overlap between the two sectors. Participants agreed that school-based platforms provide a particularly valuable opportunity for progress toward shared goals. School-based programming is considered by the soil-transmitted helminthiasis and schistosomiasis control communities to be a cost-effective method of distributing anthelmintic drugs to target groups within an established government structure. School-based WASH has received increased attention in recent years [31], and impact has been demonstrated in health, education, economic, and gender equity outcomes [32]–[34]. Soil-transmitted helminthiasis control has significant educational outcomes, as chronic helminth infections impede cognitive development and cause stunting, thus delaying school entry and grade progression. School health and nutrition programs, coordinated largely by the education sector and including both deworming and WASH improvement for increased educational attainment, have been internationally recognized platforms for more than a decade (see: http://www.freshschools.org/pages/default.aspx). An opportunity exists for both the NTD and WASH sectors to further coordinate activities within the specific, regulated environment of the school system. Marginal inputs required from each sector are low, and the mutual benefits attractive. For WASH sector civil society actors and donors, demonstrating health impact beyond the reduction of diarrheal disease may be advantageous for advocacy among donors and collaborating health institutions. For the NTD sector, facilitating improved WASH in schools ensures an environment that decreases the risk of NTD transmission among school-aged children, who represent a key risk group. For both, collaboration *via* school-based platforms opens up new potential funding sources and implementation partners.

Another cross-cutting opportunity is greater coordination at the district (sub-national) level. Both sectors currently engage in district-level activities, and context-specific institutional knowledge that contributes to program operation is often concentrated at the district level. The NTD sector already has strong district-level operational capacity, as MDA is often overseen by district- or local-level health offices. WASH service delivery is primarily managed by government actors and increasingly decentralized to the district level, while many WASH NGOs and district-level governments support community-based approaches, such as community-led total sanitation (CLTS) [35]. District-level governments, in most countries, coordinate the provision of water supply. The strong presence of both sectors at the district level could provide an opportunity for collaboration and coordination.

## Conclusion and Ways Forward

There is now considerable momentum around defining priorities and activities to achieve the WHO 2020 roadmap goals [6]. A number of networks are already in place to coordinate NTD control activities. These various coordinating bodies offer complementary skills for harmonizing efforts across the NTD control programs and synchronizing various cross-sectoral discussions with government stakeholders, drug donation programs, donors, NGOs, and academia, to set priorities for operational research and policies necessary for achieving agreed targets. Amidst this momentum toward sustainable NTD control, there is potential for expanded efforts to increase global access to water and sanitation, as insufficient access is of importance to both the WASH and the NTD sectors. The MDG for drinking water was met in 2010, but this overall achievement fails to reflect national-, regional-, and district-level disparities and says little about sustainability of realized gains. Challenges remain in reducing these disparities and in achieving increased global sanitation coverage, for which the MDG will not be met, as well as increasing good hygiene behaviors. Though hygiene is inextricably linked to disease transmission and other health outcomes, there are currently no global hygiene targets that allow for estimating progress. Post-2015 MDG targets for water and sanitation are currently being developed, with input from a network of WASH sector stakeholders.

The purpose of the Seattle two-day roundtable discussion was not to establish a new coordinating network, but to bring the WASH and NTDs sectors together to share experiences, and review challenges and opportunities for collaboration. Participants decided to continue to operate as a loose affiliation rather than establish any additional formal working groups or organizations. Discussions and decisions will be relayed to the WASH subgroup of the WHO's NTD control network. We hope that continued discussions will constitute an evolving framework for enhanced inter-sectoral collaboration, communication, and coordination at the global, regional, national, and local levels. Follow-up meetings are currently being discussed to more fully elaborate specific opportunities in mapping and data collection, as well as donor engagement. We are aware that coordinated action will be more important than continued discussion. There are a number of current activities that constitute action, but more are needed. Advocacy plans are currently in development with support from the advocacy, communications, and policy working group. Systematic reviews of the evidence base for WASH impact on several NTDs are forthcoming, and preliminary results have already been incorporated into trachoma control program meetings. A WASH-NTD manual and distance-learning course, intended to provide NTD targeting and impact information to WASH practitioners, and incorporating wide WASH and NTD sector input, is currently being developed, and will be available by December 2013. The Task Force for Global Health has recently announced a US$ 28.8 million grant from the Bill & Melinda Gates Foundation titled “Filling the gaps – operational research to ensure the success of the NTD control and evaluation programs” that will be overseen by the newly established NTD Support Center (NTD-SC). Several participants from the Seattle roundtable have participated in the NTD-SC stakeholder meetings to define priority research areas, including WASH, for the control of soil-transmitted helminthiasis, schistosomiasis, trachoma, lymphatic filariasis, and onchocerciasis.

Persistent challenges to collaboration between the WASH and NTD sectors must be acknowledged and confronted. To overcome barriers, the benefits of collaboration for each sector need to be clearly established and articulated, and common goals institutionalized in global and national policies and progress indicators. Sector-wide coordination and program integration may not be feasible ways forward within the current policy climate and existing funding structures, but strategic areas for sectoral and partner-level coordination, collaboration, and communication have been identified. These collaborations may help to more effectively and cost-effectively enhance progress toward each sector's primary goals, and toward an overall shared vision of “disease-free communities that have adequate and equitable access to water and sanitation services, and that practice good hygiene.” The opportunities described here provide guidance on actionable next steps between the two sectors. In our view, seizing these opportunities to engage in collaborative programming and research, in order to further generate learning, is the most productive step forward.
